# The correlation of the intestinal with pharyngeal microbiota in early neonates

**DOI:** 10.3389/fmicb.2023.1225352

**Published:** 2023-08-03

**Authors:** Xuejuan Wang, Zhiying Shao, Minrong Zhu, Bingjie Li, Mingyu You, Xiaoqing Chen

**Affiliations:** ^1^Department of Neonatal, Shanghai Pudong New Area Health Maternal and Child Health Hospital, Shanghai, China; ^2^Department of Pediatric, The First Affiliated Hospital of Nanjing Medical University, Nanjing, China

**Keywords:** intestinal microbiota, oropharyngeal microbiota, gut-lung axis, 16S rRNA sequencing, neonate

## Abstract

**Introduction:**

The gut-lung axis has long been recognized as an important mechanism affecting intestinal and lung immunity. Still, few studies have examined the correlation between the intestinal and pharyngeal microbiota in early neonates, especially when feeding patterns are one of the main drivers of microbiota development.

**Methods:**

To explore the composition and function of intestinal and pharyngeal microbiota and to analyze the effect of limited formula feeding on the initial microbiota colonization in early full-term neonates, we characterized the stool and oropharyngeal microbiota of 20 healthy full-term newborns sampled on days 0 and 5–7 after birth using 16S rRNA gene sequencing. Based on the sequencing results, a comparison was made of the compositions and functions of the intestinal and oropharyngeal microbiota for analysis.

**Results and discussion:**

At the phylum level, *Firmicutes, Actinobacteria, Proteobacteria*, and *Bacteroidetes* were the most abundant in both niches. At the genus level, the species of pioneer bacteria were rich in the intestine and oropharynx but low in abundance on day 0. On days 5–7, *Bifidobacterium* (25.40%) and *Escherichia-Shigella* (22.16%) were dominant in the intestine, while *Streptococcus* (38.40%) and *Staphylococcus* (23.13%) were dominant in the oropharynx. There were eight core bacteria genera in the intestine and oropharynx on days 5–7, which were *Bifidobacterium, Escherichia-Shigella, Staphylococcus, Streptococcus, Bacteroides, Parabacteroides, Rothia*, and *Acinetobacter*. As indicated by PICRUSt analysis, on days 5–7, the intestinal microbiota was more predictive than the oropharyngeal microbiota in transcription, metabolism, cell motility, cellular processes and signaling, and organismal system function in the KEGG pathway. Compared to exclusive breastfeeding, limited formula feeding (40–60%) had no significant effect on the neonatal intestinal and oropharyngeal microbiota composition during the initial colonization period. Our results suggest that the initial colonization of microbiota is closely related to the ecological niche environment in the intestine and oropharynx, with their core microbiota being closely correlated. We found that early limited formula feeding could not significantly affect the initial colonization of microbiota in the intestine and oropharynx.

## 1. Introduction

It is well acknowledged that the human microbiome serves as an important mediator of health and disease. The microbes from different body sites can have different community compositions and functions, and cross-niche connections between microbiota represent an unstudied influence on the infant microbiota (Grier et al., 2018). Studies have revealed that the microbiota in distal body sites may have a direct or indirect effect on proximal organs, as manifested in the evidence that intestinal microbiota can affect lung immunity via the gut-lung axis (Reyman et al., 2021; Mindt and DiGiandomenico, 2022; Stevens et al., 2022). The gut-lung axis, typically characterized by cross-niche microbiota interactions, has been extensively studied. On the one hand, intestinal microbial components and metabolites affect the development of lung diseases through the signaling pathways that regulate immune responses; on the other hand, lung diseases, especially infectious diseases, can cause dysbiosis to affect the intestine through immune regulation (Dang and Marsland, 2019). However, the majority of studies have focused on late neonates, infants, or older children, resulting in few studies on the initial microbiota of early neonates (Powell et al., 2019; Chiu et al., 2020; Coffey et al., 2020). Given the importance of obtaining and establishing a healthy microbiota for the symbiotic host-microbiota relationship, it is necessary that a better understanding of the initial microbiota be acquired.

The intestine, the most enriched site of the human microbiota, is easily sampled, making it widely studied. On the other hand, studies on the lung are currently limited because of its low biomass and difficulty of sampling. Further, the sampling is particularly constrained in healthy newborns due to ethical factors and parental compliance (Hufnagl et al., 2020; Stricker et al., 2022). The density and diversity of the airway microbiome are known to vary with the location within the airway. The oropharynx has the highest density of microbiota in the upper and lower respiratory tract and, therefore representative of the airway microbiome (Ma et al., 2021).

Investigating the composition and characteristics of oropharyngeal microbiota is one of the common approaches to exploring the microbiota of the respiratory tract. In the current study, we used 16S rRNA sequencing to characterize the colonization and distribution of the intestinal and oropharyngeal microbiota in healthy early neonates. The aim was to elucidate the timing of the initial action of the gut-lung axis and establish a dynamically balanced microbiota early in the colonization of the neonates carrying commensal bacteria so that we could identify the targets for the primary prevention and interventions of their respiratory disease.

Colostrum was expected to be one of the sources of the first microbiota in the intestine and oropharynx in early neonates, as indicated by previously reported evidence that the inoculation of microbiota from breast milk to the infant's intestine mainly occurred during the colostrum period (Qi et al., 2022). The problem, however, was that a number of mothers tended to have an inadequate supply of breast milk in the early postpartum period or had to temporarily add a partial supplement of formula to their newborns as a feeding transition due to certain disease statuses. Therefore, we also examined the effect of early limited formula feeding on the initial intestinal and oropharyngeal microflora composition in early neonates.

## 2. Methods

### 2.1. Study design and population

A total of 20 healthy term infants were enrolled who were delivered between September 2021 and February 2022 at Shanghai Pudong New Area Maternal and Child Health Hospital. The inclusion criteria were as follows: (1) 37 w ≤ gestational age < 42 w, BW ≥ 2,500 g, vaginal delivery; (2) singleton; (3) Being healthy during pregnancy and without maternal history of smoking, alcohol abuse, and illicit drug use; (4) No perinatal risk factors for infection and no history of antibiotic application during the perinatal period; and 5) Both the mother and newborn without a history of using probiotic or prebiotic preparation during the perinatal period. The exclusion criteria were as follows: (1) A history of asphyxia and intracranial hemorrhage at the birth of the newborn; (2) The presence of congenital malformations or inherited metabolic disorders in the newborn; (3) Newborn with hyperbilirubinemia or any other new-onset disease during the study period; (4) Incomplete collection of paired samples or incomplete data of important clinical information; and (5) Withdraw or refusal by the guardian to cooperate in the middle of the process. Of the 1,262 newborns screened, 20 met the inclusion and exclusion criteria.

The current study, approved by the Committee of Medical Ethics of Shanghai Pudong New Area Maternal and Child Health Hospital (Approval #: 20210615B), was registered in the Chinese Clinical Trial Registry (Registration #: ChiCTR2200056782). Each participant's legal guardian submitted an informed consent.

### 2.2. Collection, processing, and culture of samples

Fecal and oropharyngeal swab samples were collected by a well-trained pediatrician on day 0 and day 5–7, respectively. The first meconium of 3–5 g and fresh feces the newborn excreted naturally on day 5–7 were placed in sterile lyophilization tubes. Within 4–6 h postnatal and on day 5–7, oropharyngeal samples were collected with sterile oropharyngeal swabs with a tongue depressor to avoid contamination from the surrounding items by an experienced clinician. Three samples were collected from each newborn at a time. A consistent methodology was adopted for the storage and processing of all samples. The samples were stored at −40°C before being transferred to the temperature of −80°C within 1 week until DNA extraction.

### 2.3. Bacterial DNA extraction

Total genomic DNA was extracted from the fecal and oropharynx swab samples using E.Z.N.A™ Mag-Bind Soil DNA Kit (OMEGA, M5635-02), according to the manufacturer's instructions. The extracted DNA was quantified with Qubit 3.0 (Life, Q10212).

### 2.4. PCR method and sequencing

The bacteria genomic DNA was amplified twice with the primers of 341F (5'-CCTACGGGNGGCWGCAG-3') and 805R (5'-GACTACHVGGGTATCTAATCC-3') based on the V3-V4 variable region of the 16S rRNA gene. The PCR amplification products were examined for quality by electrophoresis on a 2% agarose gel before being sequenced using the Illumina MiSeq^TM^/Hiseq^TM^ platform for library construction.

### 2.5. Bioinformatics analysis

The raw sequences obtained from sequencing [Sangon Biotech (Shanghai) Co., Ltd (China)] were quality-controlled and optimized; the optimized sequences were clustered into operational taxonomic units (OTU) at 97% similarity level using Usearch (version 11.0.667) so that the OTU representative sequences were obtained. The representative sequences were picked for each OTU to be compared using the RDP classifier (version 2.12) based on the RDP database (http://rdp.cme.msu.edu/misc/resources.jsp) to annotate the taxonomic information for each representative sequence. To compute the alpha diversity, the OTU table was rarified, and the Shannon index and Chao1 estimator was calculated. The abundance of species in the samples was counted at the phylum and genus levels before performing the clustering analysis.

### 2.6. Clinical data collection

The clinical data were derived from the electronic medical record review, which included the physical examination at enrollment and the rest of hospitalization. Demographic characteristics included gestational age, birth weight, sex, delivery mode, Apgar scores at 1, 5, and 10 min, antibiotic exposure, probiotic or prebiotic exposure, maternal weight add, and maternal age.

### 2.7. Statistical analysis

The analyses were performed on SPSS 26 software, and the R 3.6.0 software was used to produce Venn diagrams of species distributions and the box line plots of variance tests for Chao1 and Shannon indices. Based on Bray Curtis distances, the principal component analysis (PCA) was used to illustrate the beta diversity between groups. GraphPad Prism 8.0 software was used for plotting the relative abundance histograms and the core microbiota double Y-axis graphs, and PICRUSt 1.1.4 software was used to predict the functional potential of the intestinal and oropharyngeal microbiota. STAMP 2.1.3 software was applied to the comparing of the abundance of sample functions between S2 and T2 groups and the differential taxa between feeding groups.

The normally distributed continuous variables were reported as means ± standard deviations, using two independent samples *t*-test, while Welch's *t*-test was used when the variables were not equal. The non-normally distributed continuous variables were expressed as the median (Q1, Q3) and tested using non-parametric tests. Comparisons were made between two groups using Mann-Whitney U tests, and for comparing two groups with unequal number variances, the Welch's *t*-test was used. The differentially abundant taxa were identified with the LefSe tool. Between groups, similarity was analyzed using analysis of similarities (ANOSIM analysis) along with the PERMANOVA test. The categorical data were presented as *n* (%), and the χ^2^ test or Fisher's exact tests were used for analysis. Bilateral *p* < 0.05 was considered to be statistically significant.

## 3. Results

### 3.1. Characteristics of the study population

**Figure 1:**
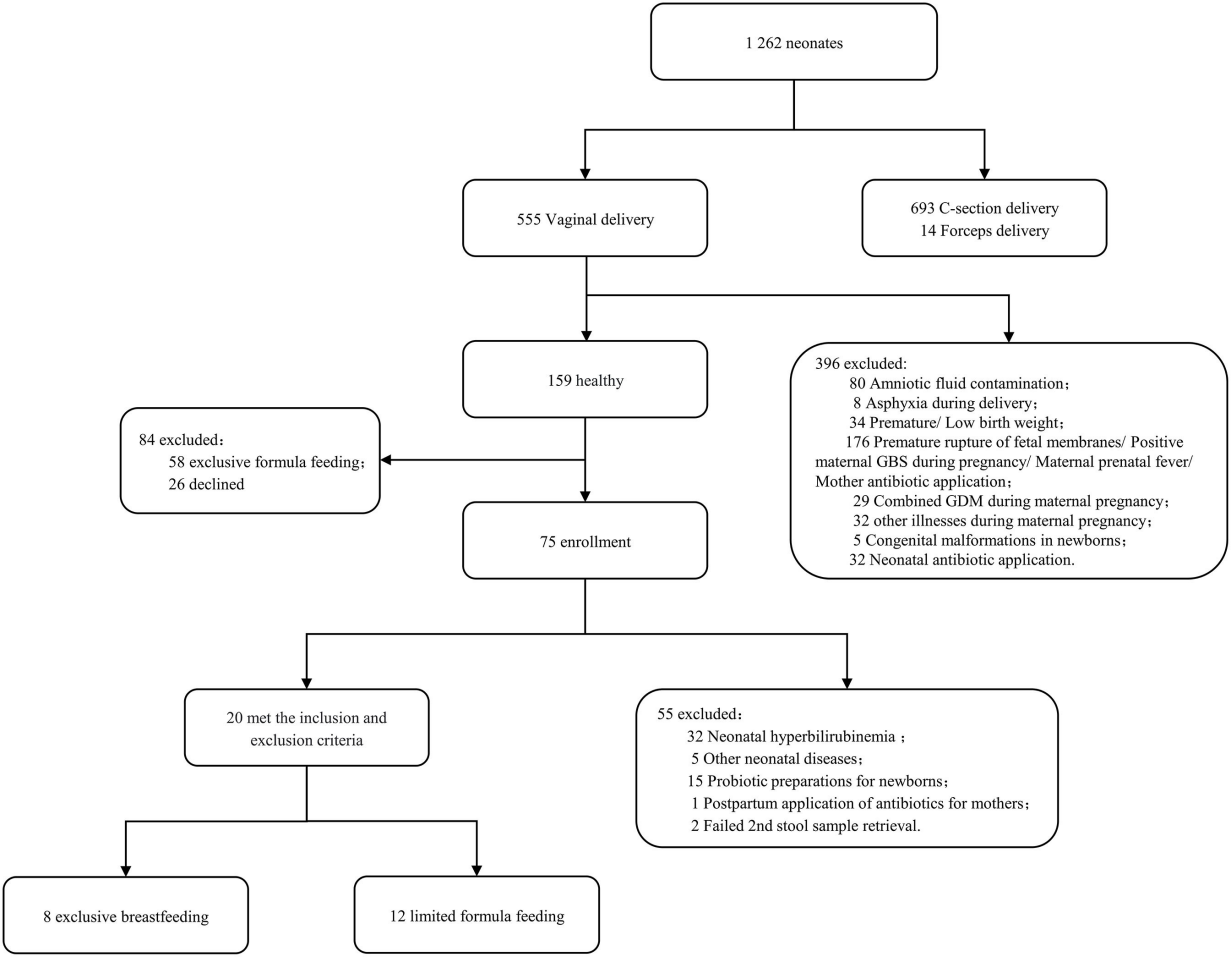
The flowchart of participant enrollment.

A total of 20 neonates were enrolled in this study. As indicated in Figure 1, all participants were born out of vaginal delivery, with their Apgar scores being 10 for 1, 5, and 10 min. According to different sampling sites and sampling time points, the samples were divided into four groups: stool on day 0 (S1), oropharyngeal swab on day 0 (T1), stool on day 5–7 (S2), and oropharyngeal swab on day 5–7 (T2). A total of 40 pairs of stool and oropharyngeal swab samples were collected from the 20 neonates, which contained detectable bacterial rRNA without exception. The clinical characteristics of the mother-neonate pairs are shown in Table 1.

**Table 1:** Clinical characteristics of 20 neonates.^a^^,^^b^

**Characteristics**	**Exclusive breastfeeding (*n* = 8)**	**Limited formula feeding (*n* = 12)**	***p*-value**
GA, days	276.63 ± 8.09	278.25 ± 3.62	0.607
BW, g	3322.50 ± 443.26	3516.25 ± 337.36	0.281
Male, *n* (%)	6 (75)	10 (83.33)	1.000^c^
**Apgar score**			
1-min	10	10	1.000
5-min	10	10	1.000
10-min	10	10	1.000
Vaginal delivery, *n*	8 (100)	12 (100)	1.000
Antibiotic exposure, *n*	0	0	1.000
Probiotic/Prebiotic exposure, *n*	0	0	1.000
Maternal Weight add, kg	13.00 ± 3.74	15.83 ± 3.738	0.114
Maternal Age, y	29.38 ± 4.24	30.00 ± 4.94	0.625

a A, gestational age; BW, birth weight.

b Data are presented as *n* (%) or mean (±SD) unless otherwise stated.

c Fisher's precision probability test.

### 3.2. High-throughput sequencing situation

**Figure 2:**
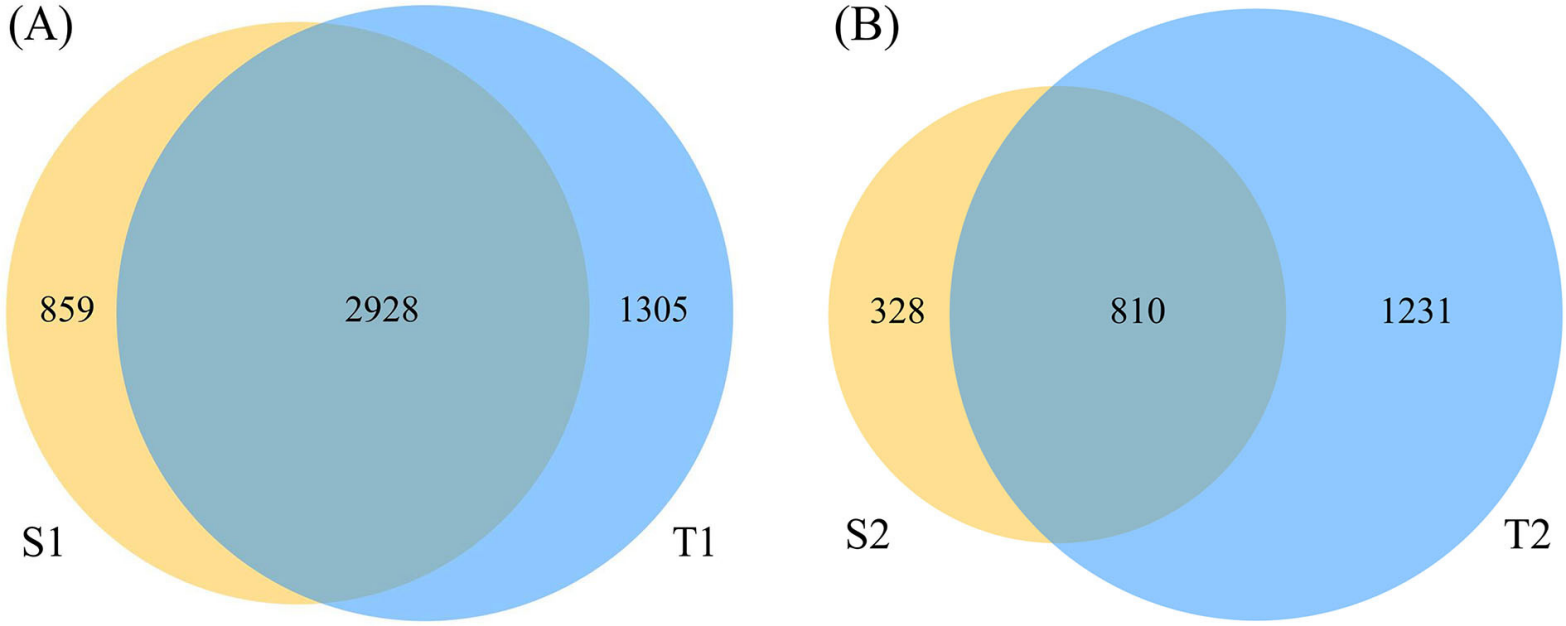
The Venn diagram of operational taxonomic units (OTU) abundance in each group. The sequencing reads shared by S1 and T1 was 2,928 **(A)**, and shared by S2 and T2 was 810 **(B)** (S1: stool on day 0; T1: oropharyngeal swab on day 0; S2: stool on day 5–7; T2: oropharyngeal swab on day 5–7).

A total of 80 samples were derived from the 20 neonates, from which 5,099,357 sequencing reads were obtained. After the filtering process, 20% of the sequences were removed, leaving 4,031,740 final valid sequences for subsequent analysis, with an average base length of 421.99 ± 6.07 bp. The sequence depth was sufficient to obtain a high degree of sequence coverage for all samples (mean: 0.998; median: 0.999; range: 0.988–0.999). More OTUs were detected in the oropharynx than in the intestine. On day 0, the percentage of OTUs shared by the intestine and oropharynx was 57.50%. On day 5–7, the percentage of OTUs shared between the intestine and oropharynx was 34.19%; the OTUs shared by the two decreased steadily with the time of birth (Figure 2).

### 3.3. Comparison of the microbial diversity in different ecological niches

#### 3.3.1. Alpha diversity

**Figure 3:**
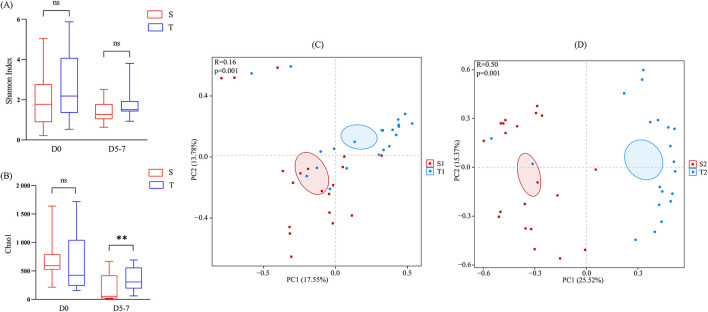
Comparison of the microbial diversity between the intestine (red) and oropharynx (blue). **(A, B)** The Chao1 and Shannon index was shown as estimators. On day 0 and day 5–7, no significant difference was observed in the Shannon index between the intestine and oropharynx **(A)**. On day 0, the Chao1 index between the intestine and oropharynx was not statistically significant, while it was higher in the oropharynx than in the intestine, with a statistically significant difference on day 5-7 **(B)**. The Mann-Whitney U test was used between every two groups. ns *p* > 0.05, **p < 0.01 (S: stool; T: oropharyngeal swab). **(C, D)** Principal component analysis (PCA) at the OTU level of each group. The horizontal and vertical axes indicate the two selected principal component axes and the percentages indicate the value of the degree of the differences in sample composition by the principal components; each sign represents an individual sample, and the points of different colors or shapes indicate the samples of different groupings. The closer the points of the two samples, the higher the degree of similarity in the species composition of the two samples. The distance between S2 and T2 **(D)** was further than S1 and T1 **(C)**. The PERMANOVA test was used between each two groups.

To assess the differences in the diversity and abundance of intestinal and oropharyngeal microbiota, we compared the Shannon and Chao1 indices. On day 0, the differences in the Shannon index (*p* = 0.3408) and Chao1 index (*p* = 0.1918) between the intestine and oropharynx were not statistically significant. On day 5-7, no significant difference was observed in the Shannon index between the intestine and oropharynx (*p* = 0.1417), while the Chao1 index was higher in the oropharynx than in the intestine (*p* = 0.0051), with a statistically significant difference (Figures 3A, B).

#### 3.3.2. Beta diversity

The ANOSIM analysis results showed that the between-group differences of the intestinal and oropharyngeal microbiota were greater than those of the within-group at the two time points and that the subgroups were comparable (Figures 3C, D). To further compare the differences in the overall microbiota composition of the intestine and oropharynx, PCA analysis was performed based on OTU levels. PERMANOVA analysis showed significant differences in the composition of the intestinal and oropharyngeal microbiota on day 0 (R^2^ = 0.074, *p* = 0.001), and the differences increased up to day 5–7 (R^2^ = 0.193, *p* = 0.001).

### 3.4. Comparison of the microbial structure of the newborns in different ecological niches

#### 3.4.1. The microbial composition of the intestine and oropharynx at the phylum level

**Figure 4:**
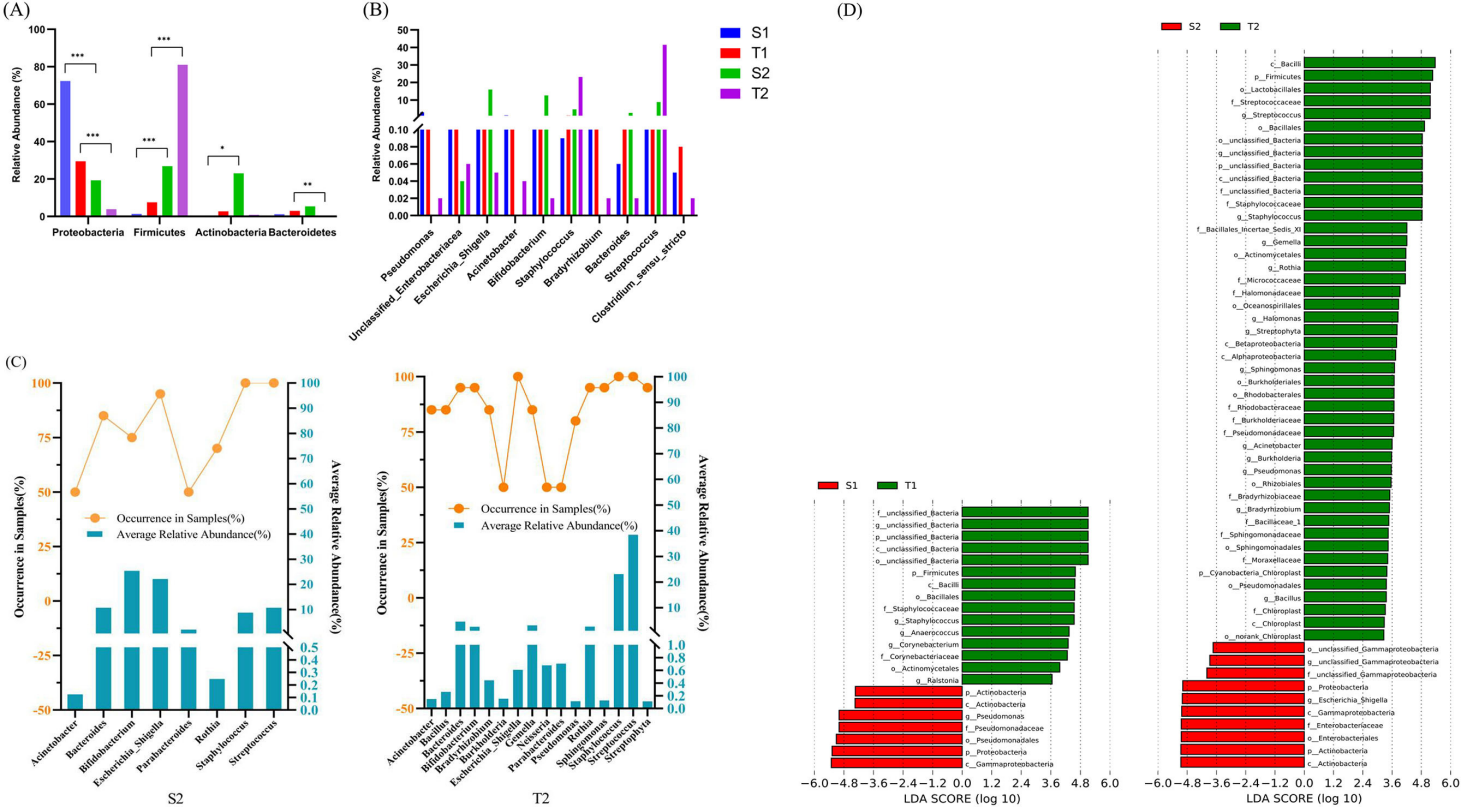
Comparison of the microbial structure of the newborns in different ecological niches. At the phylum level, both on day 0 and day 5–7, the relative abundances of *Proteobacteria, Firmicutes*, and *Actinobacteria* in the intestine and oropharynx all showed statistically significant differences, while *Bacteroidetes* showed no statistically significant difference **(A)**. At the genus level, only *Staphylococcus* and *Pseudomonas* showed statistical differences between the intestinal and oropharyngeal microbiota on day 0, but *Streptococcus, Staphylococcus, Escherichia-Shigella, Pseudomonas, Acinetobacter*, and *Bradyrhizobium* showed statistical differences on day 5–7 (all with *p* < 0.05) **(B)**. The Mann-Whitney U test was used between every two groups. *, **, and *** represent *p* ≤ 0.05, ≤0.01, and ≤0.001, respectively. **(C)** The core microbiota in the intestine and oropharynx at the genus level on day 5-7. Approximately 8 core microbiotas were observed in the intestine, while 16 core microbiotas were observed in the oropharynx. There were 8 core microbiotas shared by both, such as *Bifidobacterium, Escherichia-Shigella, Staphylococcus, Streptococcus, Bacteroides, Parabacteroides, Rothia*, and *Acinetobacter*. **(D)** Distinct bacterial taxa for the microbiota in the intestine and oropharynx at different time points that were identified by the LEfSe analysis. Green shaded areas indicate microbe orders to better describe the oropharyngeal microbiome from both time points of the neonates; red shaded areas indicate microbe orders to better describe the fecal microbiome from both time points of the neonates; The prefixes “p,” “c,” “o,” “f,” and “g” represents the annotated level of phylum, class, order, family, and genus; the genera with a linear discriminant analysis (LDA) score >3 shown for the data sets. The higher the LDA score, the greater the influence of species abundance on the difference.

The analysis of the main dominant phyla at both sites—*Firmicutes, Actinobacteria, Proteobacteria*, and *Bacteroidetes* (Figure 4A)—showed that on day 0, the relative abundances of *Proteobacteria* were 66.95 and 29.57% in the intestine and oropharynx (*p* = 0.0001), respectively, with *Firmicutes* 4.83% and 11.67% (*p* = 0.0144), *Actinobacteria* 5.64% and 3.56% (*p* = 0.0256), and *Bacteroidetes* 3.74% and 5.43% (*p* = 0.3942), respectively. On day 5-7, the relative abundance of *Proteobacteria* was 26.07% and 4.84% in the intestine and oropharynx (*p* = 0.0036), respectively, with *Firmicuria* 30.07% and 68.09% (*p* = 0.0010), *Actinobacteria* 29.93% and 5.64% (*p* = 0.0256), and *Bacteroidetes* 12.96% and 5.56% (*p* = 0.2976), respectively.

When compared with day 0, the relative abundance of *Firmicutes* and *Actinobacteria* increased significantly (*p* < 0.0001, *p* = 0.0207) while that of *Proteobacteria* decreased substantially in the intestine (*p* = 0.0001) on day 5-7, producing statistically significant differences. Similarly, the relative abundance of *Firmicutes* increased significantly (*p* < 0.0001), while that of *Proteobacteria* and *Bacteroidetes* decreased significantly in the oropharynx (*p* = 0.0001, *p* = 0.0012), showing statistically significant differences.

#### 3.4.2. The microbial composition of the intestine and oropharynx at the genus level

On day 0, the intestinal microbiota was mainly composed of *Pseudomonas* (19.45%), *unidentified_Enterobacteriaceae* (13.42%), *Escherichia-Shigella* (10.39%), *Acinetobacter* (7.91%), and *Bifidobacterium* (4.13%). Meanwhile, the oropharyngeal microbiota included *Staphylococcus* (6.98%), *unidentified_Enterobacteriaceae* (6.90%), *Bradyrhizobium* (6.06%), *Acinetobacter* (2.07%), and *Bacteroides* (1.68%). On day 5-7, the intestinal microbiota was mainly composed of *Bifidobacterium* (25.40%), *Escherichia-Shigella* (22.16%), *Bacteroides* (10.76%), *Streptococcus* (10.72%), and *Staphylococcus* (8.78%), while the oropharyngeal microbiota had *Streptococcus* (38.40%), *Staphylococcus* (23.13%), *Bacteroides* (4.64%), *Gemella* (3.13%), and *Bifidobacterium* (2.63%).

Of the top ten genera with average relative abundance, only *Staphylococcus* and *Pseudomonas* showed statistically significant differences between the intestinal and oropharyngeal microbiota on day 0 (*p* = 0.001, 0.005). By day 5–7, statistically significant differences were observed between the intestinal and oropharyngeal microbiota in terms of *Streptococcus, Staphylococcus, Escherichia-Shigella, Pseudomonas, Acinetobacter*, and *Bradyrhizobium* (all with *p* < 0.05), with an increase in differential genera (Figure 4B).

The core microbiota was defined as the genus present in ≥ 50% and average relative abundance above 10% of the samples in each group (the samples of the intestine and oropharyngeal swab) (Bach et al., 2020). On day 5–7, 8 core microbiotas were observed in the intestine, and 16 core microbiotas, in the oropharynx, with 8 core microbiotas shared by both, such as *Bifidobacterium, Escherichia-Shigella, Staphylococcus, Streptococcus, Bacteroides, Parabacteroides, Rothia*, and *Acinetobacter* (Figure 4C).

#### 3.4.3. Community structure differences between the intestinal and oropharyngeal microbiota

The analysis of the microbiota of fecal and oropharyngeal swab samples using the LEfSe tool to detect potentially significant differences in the relative abundance of the intestine and oropharyngeal microbiota revealed that there were significant differences between S1 and T1 groups in five genera. *Pseudomonas* were more abundant in S1, and *Staphylococcus, Anaerococcus, Corynebacterium*, and *Ralstonia* were more abundant in T1. The results further showed that the number of different bacteria genera increased significantly in S2 and T2 groups; the *unclassified_Gammaproteobacteria* and *Escherichia_Shigella* were more abundant in S2 than in T2; *Streptococcus, Staphylococcus, Gemella, Rothia, Halomonas, Streptophyta, Sphingomonas, Acinetobacter, Burkholderia, Pseudomonas, Bradyrhizobium*, and *Bacillus* were more abundant in T2 than in S2 (Figure 4D).

### 3.5. The functional predictive analysis of the intestinal and oropharyngeal microbiota

**Figure 5:**
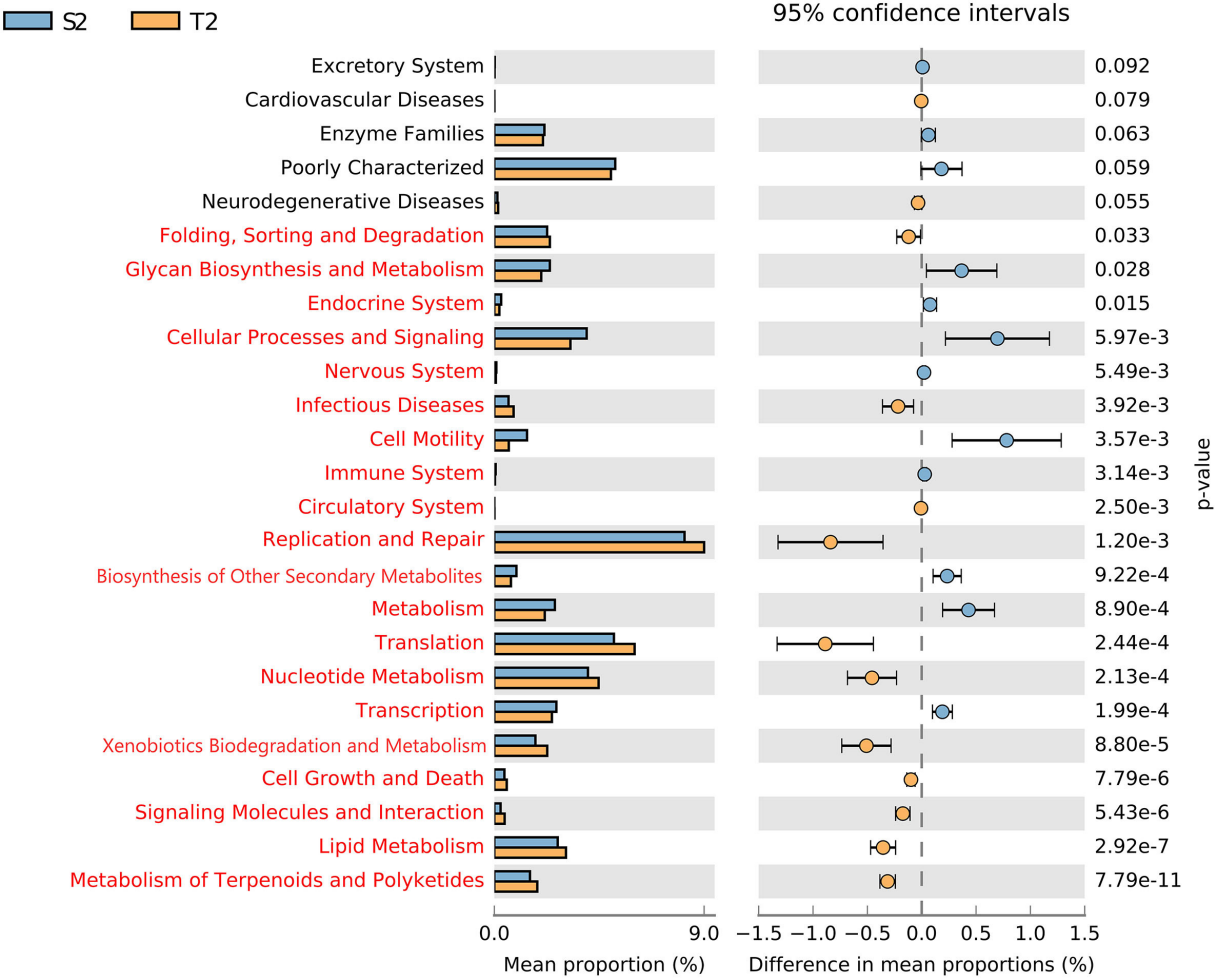
Different analyses of the metabolic function enrichment in S2 and T2. The left indicates the proportion of the relative abundances of different microbiota functions in the two groups; the middle indicates the proportion of differences in the relative abundance of microbiota functions within the 95% confidence interval; the far right shows *p*; *p* < 0.05 indicates a significant difference, marked in red.

To determine the function of the initial microbiota, the functional potential of the intestinal and oropharyngeal microbiota was predicted on PICRUSt 1.1.4. On STAMP 2.1.3 software, the functional abundance of S2 and T2 were compared. Through the analysis of the metabolic pathways at the Kyoto Encyclopedia of Genes and Genomes (KEGG) secondary taxonomic level, it was found that the intestinal microbiota carried a higher gene function in transcription; metabolism; biosynthesis of other secondary metabolites; immune system; cell motility; nervous system; cellular processes and signaling; glycan biosynthesis and metabolism, and lower gene function in metabolic pathways such as metabolism of terpenoids and polyketides; lipid metabolism; signaling molecules and interaction; cell growth and death; xenobiotics biodegradation and metabolism; nucleotide metabolism; and translation other than the oropharyngeal microbiota (all with *p* < 0.05, Figure 5).

## 4. Discussion

The colonization and progressive diversification of primary microorganisms in an infant's gut play an important role in establishing a symbiotic system of host-microbe interactions (Shi et al., 2018). In the current study, we used 16S rRNA gene sequencing technology to detect the microbiota composition and differences in the intestine and oropharynx of full-term healthy newborns. The results showed more microbiota categories in the oropharynx than in the intestine. The microbial diversity was similar in the intestine and oropharynx during this research period, while the microbiota was richer on day 5–7 in the oropharynx, consistent with the findings of Yang et al. (2021). The results of the PCA analysis showed a more pronounced separation between different samples of the intestine and oropharynx, suggesting a large individual difference between samples within the group, which may be related to the genetic and environmental factors of the host.

The microbial composition analysis showed that *Firmicutes, Actinobacteria, Proteobacteria*, and *Bacteroidetes* were the most abundant taxa in the intestine and oropharynx at the time points of both, which was consistent with the findings of Bach et al. (2020) and Ibironke et al. (2020). However, the composition of the microbiota produced a significant difference between the two niches and time points. In the intestine, the relative abundance of *Proteobacteria* was highest on day 0; the relative abundance of *Proteobacteria, Firmicutes, Actinobacteria*, and *Bacteroidetes* were approximately equal on day 5–7. In the oropharynx, the relative abundance of *Proteobacteria* was highest on day 0, followed by *Firmicutes*; by day 5–7, *Firmicutes* was the absolutely dominant phylum. At the genus level, the two ecological niches were rich in species and low in the abundance of pioneer genera on day 0, with few differential genera; the two ecological niches steadily showed their different specific taxa on day 5–7, with *Bifidobacterium* (25.40%) and *Escherichia-Shigella* (22.16%) predominating in the intestine. These findings were in agreement with those previously reported by Bäckhed et al. (2015). On day 5–7, *Streptococcus* (38.40%) and *Staphylococcus* (23.13%) predominated in the oropharynx, and these findings were consistent with those previously reported by Maleki et al. (2020). Furthermore, between the two niches, there existed more significantly different bacteria genera. These findings suggested that the initial microbiota could be diverse and low in abundance in the intestine and oropharynx of the early neonates and that the microbiota of each ecological niche could develop rapidly during the first week of life, with the emergence of their specific dominant microbiota, which is consistent with the findings of Pattaroni et al. (2022).

According to a previous study, the abundance of specific taxa in one body site exhibited strong associations with the community types of the others (Grier et al., 2018). In this study, all the core microbiota observed in the intestine were shared with the oropharynx in the early neonates. Thus, we hypothesized that the probiotics, which are capable of regulating the intestinal microbiota, could also regulate the respiratory microbiota and affect lung immunity. Further analyzing the shared core microbiota, we found that the core dominant bacteria differed between the intestine and the oropharynx; the intestine had *Bifidobacterium* and *Escherichia-Shigella*. *Bifidobacterium*, known to be a health-related microorganism for its role as a probiotic (Reyman et al., 2019), promotes intestinal health and protects against pathogens (Pan et al., 2021; Lin et al., 2022) and is associated with a lower incidence of respiratory infections and asthma (Wypych et al., 2019). The delay in the establishment of *Bifidobacterium* may have a significant impact on the early life and future health of infants. In the oropharynx, *Streptococcus* was the core dominant genus of bacteria. *Streptococcus* is an early oropharyngeal colonizer and plays an irreplaceable role in establishing the initial microbiota, and is characterized by a heterogeneous population that includes commensal bacteria and pathogens. The commensal streptococci can produce hydrogen peroxide to inhibit the growth of pathogenic microbiota, including methicillin-resistant *Staphylococcus* aureus and *Pseudomonas* aeruginosa in the hospital, and regulate the immune balance of colonization sites. Thus, far, researchers have explored using *commensal streptococci* as probiotics to regulate susceptibility to respiratory infections (Al-Shehri et al., 2016; Clark, 2020). In addition, *Lactobacillus*, another known probiotic, especially sIgA-bound *Lactobacillus*, has been reported to possess the potential to interact with the host gut to regulate homeostasis (Sun et al., 2019; Qi et al., 2021). The current study showed no significant difference in the relative abundance of *Lactobacillus* in the gut and oropharynx of early neonates (*p* = 0.3648, 0.1115), which may be one of the directions to explore respiratory probiotics.

It has been well recognized that feeding patterns are one of the main factors affecting the process of microbiota acquisition and establishment in early neonates (Ruiz et al., 2019; Kaan et al., 2021). For newborns, exclusive breastfeeding is the best way to get the benefits of immunity and healthy outcomes. Compared with mature milk, colostrum performs the main function of immunity (Toscano et al., 2017) and has been called “liquid gold” because it plays an irreplaceable role in the initiation of a balanced immune system and the acquisition and colonization of the initial microbiota during the early neonatal period. In the current study, however, we found that quite a number of mothers had insufficient breast milk in the early stage after delivery, and they needed partial supplementation with formula to help their newborns make a feeding transition. As shown in the Supplementary material, when compared with exclusive breastfeeding, early limited formula feeding (40–60%) during the initial colonization period had no significant effect on the composition of microbiota in the intestine and oropharynx of the early neonates, which was consistent with the findings of Bäckhed et al. (2015). Therefore, we hypothesize that the anatomical niche itself is the most important explanatory variable for the initial microbial community composition and that the environment of the niche is the main driver of the local microbiota colonization.

Our study suggested that the intestinal microbiota could perform a higher gene function in transcription, metabolism, biosynthesis of other secondary metabolites, immune system, cell motility, nervous system, cellular processes and signaling, and glycan biosynthesis and metabolism. Meanwhile, the oropharyngeal microbiota was more highly predictable in such metabolic pathways as the metabolism of terpenoids and polyketides, lipid metabolism, signaling molecules and interaction, cell growth and death, xenobiotics biodegradation and metabolism, nucleotide metabolism, and translation. It is evident that the intestinal microbiota can affect the metabolic, immune, and neurological functions of the human body, as previously established with the regulation of the intestinal microbiota that could function as one of the effective strategies for the prevention and treatment of lung diseases (Sencio et al., 2020; Wang et al., 2020). Based on the relevance and differences in the composition and function of the intestinal and oropharyngeal microbiota, the development of probiotic agents or antimicrobial therapies could offer an approach to preventing or treating respiratory diseases.

Although the inclusion criteria were strictly controlled and the intensity of formula addition was equal between the observation groups, our study still contained some limitations. One limitation arose from the relatively small number of samples. In addition, due to ethical concerns over the collection of the lower respiratory tract samples from healthy neonates, we failed to conduct a comparative follow-up of the samples. Finally, the analysis was conducted only at the genus level, which could be explained by the limitation of detection techniques.

In conclusion, we succeeded in identifying the key early neonatal intestinal and oropharyngeal strain categories, which we believe can facilitate the establishment of a normal base model for subsequent intervention studies. Furthermore, we confirmed that early transition feeding with moderate amounts of formula did not alter the initial colonization of the intestinal and oropharyngeal microbiota.

## Data availability statement

The datasets presented in this study can be found in online repositories. The names of the repository/repositories and accession number(s) can be found below: NCBI-PRJNA975837.

## Ethics statement

The studies involving human participants were reviewed and approved by the Committee of Medical Ethics of Shanghai Pudong New Area Maternal and Child Health Hospital (Approval #: 20210615B). Written informed consent to participate in this study was provided by the participants' legal guardian/next of kin.

## Author contributions

XC designed this study. XW, ZS, and MZ collected the samples. BL and MY analyzed the data. XW and XC wrote and revised this paper. All authors have read and approved the manuscript.

## References

[B1] Al-Shehri S. S. Sweeney E. L. Cowley D. M. Liley H. G. Ranasinghe P. D. Charles B. G.. (2016). Deep sequencing of the 16S ribosomal RNA of the neonatal oral microbiome: a comparison of breast-fed and formula-fed infants. Sci. Rep. 6, 38309. 10.1038/srep38309 27922070 PMC5138828

[B2] Bach L. L. Ram A. Ijaz U. Z. Evans T. J. Lindström J. (2020). A longitudinal study of the human oropharynx microbiota over time reveals a common core and significant variations with self-reported disease. Front. Microbiol. 11, 573969. 10.3389/fmicb.2020.573969 33552004 PMC7861042

[B3] Bäckhed F. Roswall J. Peng Y. Feng Q. Jia H. Kovatcheva-Datchary P.. (2015). Dynamics and stabilization of the human gut microbiome during the first year of life. Cell Host. Microbe. 17, 690–703. 10.1016/j.chom.2015.04.004 25974306

[B4] Chiu C. Y. Chan Y. L. Tsai M. H. Wang C. J. Chiang M. H. Chiu C. C.. (2020). Cross-talk between airway and gut microbiome links to IgE responses to house dust mites in childhood airway allergies. Sci. Rep. 10, 13449. 10.1038/s41598-020-70528-7 32778700 PMC7417544

[B5] Clark S. E. (2020). Commensal bacteria in the upper respiratory tract regulate susceptibility to infection. Curr Opin Immunol. 66, 42–49. 10.1016/j.coi.2020.03.010 32416468 PMC7665980

[B6] Coffey M. J. McKay I. R. Doumit M. Chuang S. Adams S. Stelzer-Braid S.. (2020). Evaluating the Alimentary and Respiratory Tracts in Health and disease (EARTH) research programme: a protocol for prospective, longitudinal, controlled, observational studies in children with chronic disease at an Australian tertiary paediatric hospital. BMJ Open. 10, :e033916. 10.1136/bmjopen-2019-033916 32295774 PMC7200033

[B7] Dang A. T. Marsland B. J. (2019). Microbes, metabolites, and the gut-lung axis. Mucosal Immunol. 12, 843–850. 10.1038/s41385-019-0160-6 30976087

[B8] Grier A. McDavid A. Wang B. Qiu X. Java J. Bandyopadhyay S.. (2018). Neonatal gut and respiratory microbiota: coordinated development through time and space. Microbiome. 6, 193. 10.1186/s40168-018-0566-5 30367675 PMC6204011

[B9] Hufnagl K. Pali-Schöll I. Roth-Walter F. Jensen-Jarolim E. (2020). Dysbiosis of the gut and lung microbiome has a role in asthma. Semin. Immunopathol. 42, 75–93. 10.1007/s00281-019-00775-y 32072252 PMC7066092

[B10] Ibironke O. McGuinness L. R. Lu S. E. Wang Y. Hussain S. Weisel C. P.. (2020). Species-level evaluation of the human respiratory microbiome. Gigascience. 9, giaa038. 10.1093/gigascience/giaa038 32298431 PMC7162353

[B11] Kaan A. M. M. Kahharova D. Zaura E. (2021). Acquisition and establishment of the oral microbiota. Periodontol 86, 123–141. 10.1111/prd.12366 33690935 PMC8252790

[B12] Lin C. Lin Y. Zhang H. Wang G. Zhao J. Zhang H.. (2022). Intestinal 'infant-type' bifidobacteria mediate immune system development in the first 1000 days of life. Nutrients. 14, 1498. 10.3390/nu14071498 35406110 PMC9002861

[B13] Ma S. Zhang F. Zhou F. Li H. Ge W. Gan R.. (2021). Metagenomic analysis reveals oropharyngeal microbiota alterations in patients with COVID-19. Signal Transduct. Target. Ther. 6, 191. 10.1038/s41392-021-00614-3 33986253 PMC8116522

[B14] Maleki A. Zamirnasta M. Taherikalani M. Pakzad I. Mohammadi J. Krutova M.. (2020). The characterization of bacterial communities of oropharynx microbiota in healthy children by combining culture techniques and sequencing of the 16S rRNA gene. Microb. Pathog. 143, 104115. 10.1016/j.micpath.2020.104115 32135220

[B15] Mindt B. C. DiGiandomenico A. (2022). Microbiome Modulation as a Novel Strategy to Treat and Prevent Respiratory Infections. Antibiotics. 11, 474. 10.3390/antibiotics11040474 35453224 PMC9029693

[B16] Pan K. Zhang C. Tian J. (2021). The effects of different modes of delivery on the structure and predicted function of intestinal microbiota in neonates and early infants. Pol. J. Microbiol. 70, 45–55. 10.33073/pjm-2021-002 33815526 PMC8008759

[B17] Pattaroni C. Macowan M. Chatzis R. Daunt C. Custovic A. Shields M. D.. (2022). Early life inter-kingdom interactions shape the immunological environment of the airways. Microbiome. 10, 34. 10.1186/s40168-021-01201-y 35189979 PMC8862481

[B18] Powell E. A. Fontanella S. Boakes E. Belgrave D. Shaw A. G. Cornwell E.. (2019). Temporal association of the development of oropharyngeal microbiota with early life wheeze in a population-based birth cohort. EBioMed. 46, 486–498. 10.1016/j.ebiom.2019.07.034 31353293 PMC6710983

[B19] Qi C. Ding M. Li S. Zhou Q. Li D. Yu R.. (2021). Sex-dependent modulation of immune development in mice by secretory IgA-coated Lactobacillus reuteri isolated from breast milk. J. Dairy Sci. 104, 3863–3875. 10.3168/jds.2020-19437 33612242

[B20] Qi C. Zhou J. Tu H. Tu R. Chang H. Chen J.. (2022). Lactation-dependent vertical transmission of natural probiotics from the mother to the infant gut through breast milk. Food Funct. 13, 304–315. 10.1039/D1FO03131G 34889924

[B21] Reyman M. Clerc M. van Houten M. A. Arp K. Chu M. L. J. N. Hasrat R.. (2021). Microbial community networks across body sites are associated with susceptibility to respiratory infections in infants. Commun. Biol. 4, 1233. 10.1038/s42003-021-02755-1 34711948 PMC8553847

[B22] Reyman M. van Houten M. A. van Baarle D. Bosch A. A. T. M. Man W. H. Chu M. L. J. N.. (2019). Impact of delivery mode-associated gut microbiota dynamics on health in the first year of life. Nat. Commun. 10, 4997. 10.1038/s41467-019-13014-7 31676793 PMC6825150

[B23] Ruiz L. Bacigalupe R. García-Carral C. Boix-Amoros A. Argüello H. Silva C. B.. (2019). Microbiota of human precolostrum and its potential role as a source of bacteria to the infant mouth. Sci. Rep. 9, 8435. 10.1038/s41598-019-42514-16 31182726 PMC6557856

[B24] Sencio V. Barthelemy A. Tavares L. P. Machado M. G. Soulard D. Cuinat C.. (2020). Gut dysbiosis during influenza contributes to pulmonary pneumococcal superinfection through altered short-chain fatty acid production. Cell Rep. 30, 2934–2947. 10.1016/j.celrep.2020.02.013 32130898

[B25] Shi Y. C. Guo H. Chen J. Sun G. Ren R. R. Guo M. Z.. (2018). Initial meconium microbiome in Chinese neonates delivered naturally or by cesarean section. Sci. Rep. 8, 3255. 10.1038/s41598-018-21657-7 29459704 PMC5818670

[B26] Stevens J. Steinmeyer S. Bonfield M. Peterson L. Wang T. Gray J.. (2022). The balance between protective and pathogenic immune responses to pneumonia in the neonatal lung is enforced by gut microbiota. Sci. Transl. Med. 14, eabl3981. 10.1126/scitranslmed.abl3981 35704600 PMC10032669

[B27] Stricker S. Hain T. Chao C. M. Rudloff S. (2022). Respiratory and intestinal microbiota in pediatric lung diseases-current evidence of the gut-lung axis. Int. J. Mol. Sci. 23, 6791. 10.3390/ijms23126791 35743234 PMC9224356

[B28] Sun J. Qi C. Zhu H. Zhou Q. Xiao H. Le G.. (2019). IgA-targeted lactobacillus jensenii modulated gut barrier and microbiota in high-fat diet-fed mice. Front. Microbiol. 10, 1179. 10.3389/fmicb.2019.01179 31178854 PMC6542990

[B29] Toscano M. Grandi D. Grossi R. Drago E. L. (2017). Role of the human breast milk-associated microbiota on the newborns' immune system: a mini review. Front. Microbiol. 8, 2100. 10.3389/fmicb.2017.02100 29118752 PMC5661030

[B30] Wang W. Luo X. Zhang Q. He X. Zhang Z. Wang X.. (2020). Bifidobacterium infantis relieves allergic asthma in mice by regulating Th1/Th2. Med. Sci. Monit. 26, e920583. 10.12659/MSM.920583 32249275 PMC7160606

[B31] Wypych T. P. Wickramasinghe L. C. Marsland B. J. (2019). The influence of the microbiome on respiratory health. Nat. Immunol. 20, 1279–1290. 10.1038/s41590-019-0451-9 31501577

[B32] Yang S. Qiao L. Shi J. Xie L. Liu Y. Xiong Y.. (2021). Clinical study of correlation for the intestinal and pharyngeal microbiota in the premature neonates. Front. Pediatr. 9, 632573. 10.3389/fped.2021.632573 33665178 PMC7920978

