# Supplementary material for: The correlation of the intestinal with pharyngeal microbiota in early neonates
Source: Front Microbiol. 2023 Aug 3;14:1225352. doi: 10.3389/fmicb.2023.1225352 (PMC10434775; doi:10.3389/fmicb.2023.1225352)
Supplement: Supplementary file 1 [file Data_Sheet_1.pdf]

Supplementary Material

Supplementary Material:

1 Effects of different feeding patterns on the composition of the intestinal and oropharyngeal microbiota

To compare the effect of limited formula feeding on microbiota of 20 early neonates who were divided into exclusive breast feeding group (n=8, B) and limited formula feeding group (n=12, M, on 40%-60% of formula), their faecal (BS, MS) and oropharyngeal swab (BT, MT) samples, collected on day 5-7, underwent an analysis. As indicated in Table 1, no significant differences were observed between the two groups in terms of neonatal sex (*P*=1.000), gestational age (*P*=0.607), birth weight (*P*=0.281), maternal gestational weight gain (*P*=0.114) and maternal age (*P*=0.625). STAMP 2.1.3 software package was applied to the statistical analysis, and Welch's-t test,sdx to the comparison of the microbiota abundance variation between the groups. The results showed that the limited formula feeding had no significant effect on the composition of the intestinal microbiota, and that the significant different genera of oropharyngeal microbiota were rare species with low abundance; there was no significant effect on the dominant oropharyngeal genera (**Supplementary Figure**).

(A)

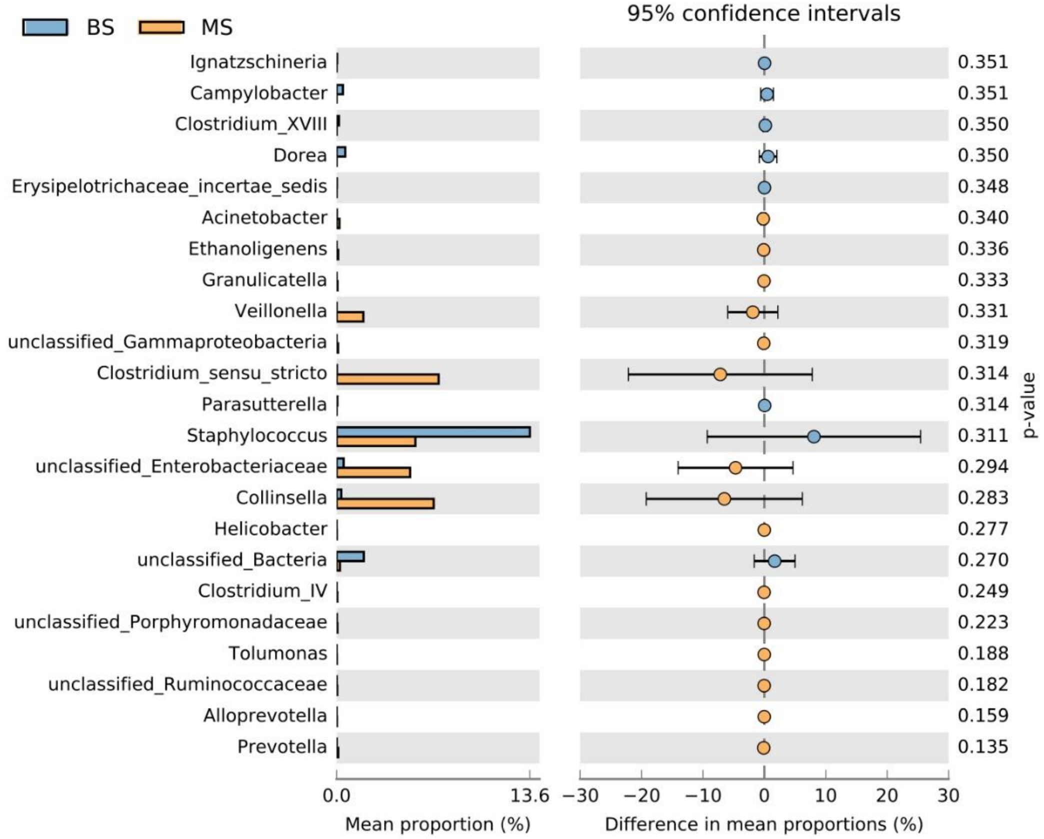
BS
MS
95% confidence intervals
Ignatzschineria
Campylobacter
Clostridium_XVIII
Dorea
Erysipelotrichaceae_incertae_sedis
Acinetobacter
Ethanoligenens
Granulicatella
Veillonella
unclassified_Gammaproteobacteria
Clostridium_sensu_stricto
Parasutterella
Staphylococcus
unclassified_Enterobacteriaceae
Collinsella
Helicobacter
unclassified_Bacteria
Clostridium_IV
unclassified_Porphyromonadaceae
Tolumonas
unclassified_Ruminococcaceae
Alloprevotella
Prevotella
0.351
0.351
0.350
0.350
0.348
0.340
0.336
0.333
0.331
0.319
0.314
0.314
0.311
0.294
0.283
0.277
0.270
0.249
0.223
0.188
0.182
0.159
0.135
p-value
0.0
13.6
−30
−20
−10
0
10
20
30
Mean proportion (%)
Difference in mean proportions (%)

(B)

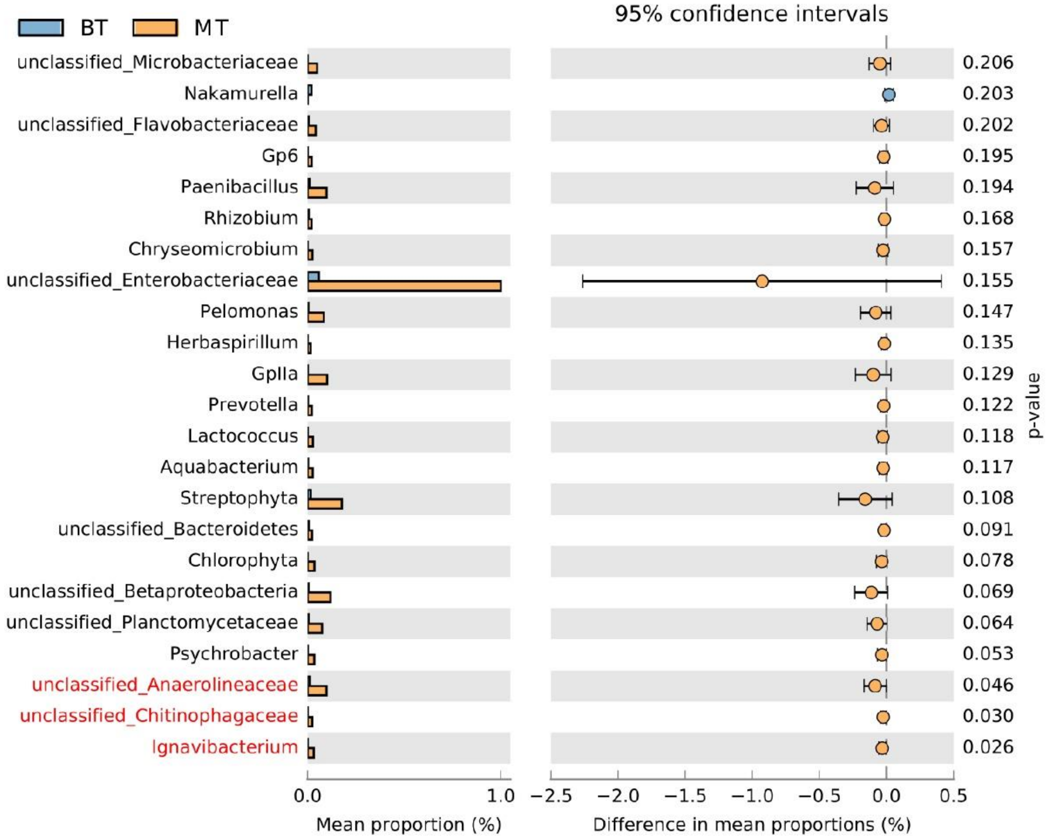
BT
MT
95% confidence intervals
unclassified_Microbacteriaceae
Nakamurella
unclassified_Flavobacteriaceae
Gp6
Paenibacillus
Rhizobium
Chryseomicrobium
unclassified_Enterobacteriaceae
Pelomonas
Herbaspirillum
GpIIa
Prevotella
Lactococcus
Aquabacterium
Streptophyta
unclassified_Bacteroidetes
Chlorophyta
unclassified_Betaproteobacteria
unclassified_Planctomycetaceae
Psychrobacter
unclassified_Anaerolineaceae
unclassified_Chitinophagaceae
Ignavibacterium
0.206
0.203
0.202
0.195
0.194
0.168
0.157
0.155
0.147
0.135
0.129
0.122
0.118
0.117
0.108
0.091
0.078
0.069
0.064
0.053
0.046
0.030
0.026
p-value
0.0
1.0
−2.5
−2.0
−1.5
−1.0
−0.5
0.0
0.5
Mean proportion (%)
Difference in mean proportions (%)

Supplementary Figure

Distribution of the relative abundance of taxa among different groups (mean relative aboundence > 0.1%). The left indicating the proportion of relative abundances of different microbiota in the two groups; the middle indicating the proportion of differences in the relative abundance of microbiota within the 95% confidence interval; the far right indicating p; $p<0.05$ indicating a significant difference, marked in red. **(A, B)** There was no significant differences were observed on the dominant genera in the intestine and oropharynx under different feeding patterns, and that the significant different genera of oropharyngeal microbiota were rare species with low abundance. (BS: stool on day 5-7, exclusive breast feeding; MS: stool on day 5-7, limited formula feeding; BT: oropharyngeal swab on day 5-7, exclusive breast feeding; MT: oropharyngeal swab on day 5-7, limited formula feeding).
